# Performance Optimization Strategies for Polymer Organic Field-Effect Transistors as Sensing Platforms

**DOI:** 10.3390/s25226891

**Published:** 2025-11-11

**Authors:** Yan Wang, Zimin Ye, Tianci Wang, Linxiao Zu, Liwen Chen

**Affiliations:** School of Health Science and Engineering, University of Shanghai for Science and Technology, Shanghai 200093, China; 232322279@st.usst.edu.cn (Y.W.);

**Keywords:** organic field-effect transistor, polymer semiconductors, carrier mobility, sensors, interface

## Abstract

Organic field-effect transistors (OFETs) have emerged as a transformative platform for high-performance sensing technologies, yet their full potential can be realized only through coordinated performance optimization. This article provides a comprehensive review of recent strategies employed in polymer OFETs to enhance key parameters, including carrier mobility (μ), threshold voltage (Vth), on/off current ratio (I_on_/I_off_), and operational stability. These strategies encompass both physical and chemical approaches, such as annealing, self-assembled monolayers (SAMs), modification of main and side polymer chains, dielectric-layer engineering, buffer-layer insertion, and blending or doping techniques. The development of high-performance devices requires precise integration of physical processing and chemical design, alongside the anticipation of processing compatibility during the molecular design phase. This article further highlights the limitations of focusing solely on high mobility and advocates a balanced optimization across multiple dimensions—mobility, mechanical flexibility, environmental stability, and consistent functional performance. Adopting a multi-scale optimization framework spanning molecular, film, and device levels can substantially enhance the adaptability of OFETs for emerging applications such as flexible sensing, bioelectronic interfaces, and neuromorphic computing.

## 1. Introduction

Organic field-effect transistors (OFETs), as core components of organic electronics, offer advantages such as facile fabrication, mechanical flexibility, and broad application potential that surpass those of conventional inorganic semiconductor devices [1]. They demonstrate considerable promise for deployment in flexible electronic technologies and intelligent sensing systems, exhibiting notable features such as low power consumption, high integration density, and exceptional flexibility [2].

Owing to specific interactions between recognition elements and target analytes, the conductivity of an OFET’s channel exhibits high sensitivity to binding events [3]. Coupled with its unique signal amplification capability, which converts weak chemical or physical stimuli into amplified electrical signals, the OFET enables high-sensitivity detection and serves as a core component in various sensing applications. The sensing mechanism essentially involves the interactions between analytes and the device, which modulate its electrical properties [4]. The performance of OFETs is typically assessed by key parameters such as carrier mobility (μ), threshold voltage (Vth), on/off current ratio (I_on_/I_off_), sub-threshold swing (SS), and operational stability [5]. These factors fundamentally determine sensor performance metrics, including sensitivity, limit of detection (LOD), selectivity, and response and recovery times. Importantly, these parameters are interdependent and involve inherent trade-offs. Consequently, successful optimization requires balancing them according to specific sensing requirements.

**Figure 1:**
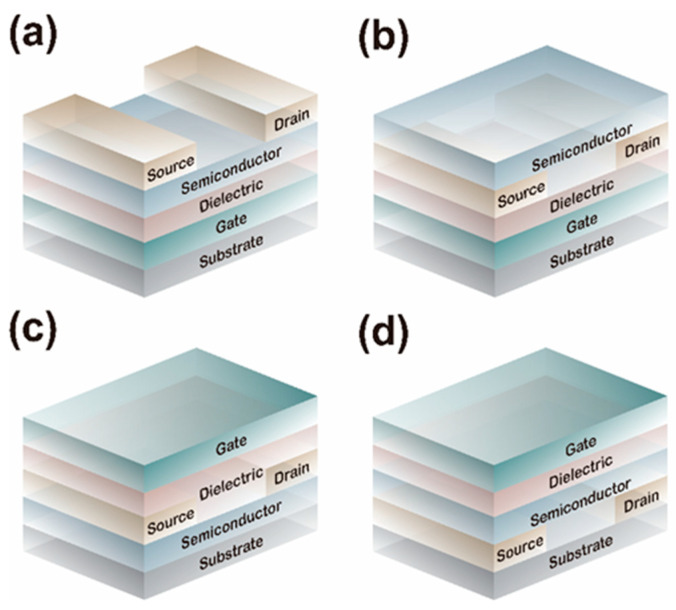
Schematic illustration of typical OFETs configurations: (**a**) bottom-gate top-contact (BG-TC); (**b**) bottom-gate bottom-contact (BG-BC); (**c**) top-gate top-contact (TG-TC); (**d**) top-gate bottom-contact (TG-BC).

The performance characteristics of an OFET are largely governed by its structural design. A typical OFET comprises a semiconductor layer, source and drain electrodes, a gate electrode, a dielectric layer, and a substrate [5,6]. Depending on the arrangement of the constituent layers, OFETs are categorized into four configurations: bottom-gate top-contact (BG-TC), bottom-gate bottom-contact (BG-BC), top-gate top-contact (TG-TC), and top-gate bottom-contact (TG-BC) structures [5,7] (Figure 1). When a gate voltage is applied, a vertical electric field forms at the surface of the dielectric layer, inducing equal and opposite charges at the interface between the dielectric layer and the organic semiconductor (OSC). This charge accumulation generates a conductive channel through which current flows between the source and drain under the applied voltage.

Early OFET research primarily focused on identifying high-performance OSCs capable of fulfilling the device’s core functions. OSCs are carbon-based optoelectronic materials composed of molecular assemblies held together by van der Waals forces, offering advantages such as mechanical flexibility and cost-effectiveness [8]. They are generally categorized into small molecules and conjugated polymers (CPs). Among these, CPs have garnered extensive attention and application in organic electronics due to their superior charge-transport characteristics, adjustable molecular structures, and multifunctional attributes such as solution processability, molecular engineering potential, flexibility, and printability [9,10]. The molecular-level characteristics of CPs directly influence the microscopic morphology (e.g., crystallinity, molecular orientation, phase separation) and electronic properties (e.g., energy level, band gap) of the resulting films [11,12]. These, in turn, profoundly impact key device parameters. A comprehensive understanding of the intricate interrelations among the polymer structure, processing conditions, morphology characteristics, and device performance—along with the development of effective physical and chemical modulation strategies—is therefore essential for enhancing polymer OFET performance and facilitating practical applications.

In recent years, advancements in understanding the intrinsic properties of polymer semiconductors and their device behavior have driven the development and widespread application of various physical and chemical strategies. These approaches are designed to optimize interfacial contact, enhance film ordering, regulate energy levels, reduce defect density, and improve environmental stability. They cover a broad spectrum of methods, including processing and post-treatment techniques such as annealing, self-assembled monolayer (SAM) modification, and buffer-layer incorporation, as well as molecular design strategies such as main-chain and side-chain engineering. Material formulation and modification through blending, doping, and related methods have also proven effective. This review systematically examines these methodologies for improving the performance of polymer OFETs, analyzes their underlying mechanisms and effects, and discusses their applications in the field of sensing, together with prospective directions for future research.

## 2. Strategies for Performance Improvement

To systematically enhance the sensing capability of OFETs, core electrical properties must be optimized at their fundamental levels. These properties are constrained not only by the inherent characteristics of the semiconductor materials but also by multiple device interfaces [13], which critically influence performance. A two-tier hierarchy fundamentally governs the key parameters of polymer OFETs. At the material level, performance is determined by the semiconductor’s intrinsic electronic structure and by film morphology variables such as conjugated main-chain orientation, side-chain packing, thin-film crystallinity, and phase-separation structure. At the device level, interface characteristics—including electrode/semiconductor contact quality and trap-state distribution at the dielectric/semiconductor interface—are equally significant. These factors collectively form the physical basis that influences sensor sensitivity, response and recovery times, LOD, and stability. Table 1 summarizes how OFET electrical properties affect sensing performance.

This section examines strategies for improvement—annealing, SAMs, dielectric-layer modification, buffer-layer insertion, main-chain and side-chain engineering, and blending and doping—and elucidates the mechanisms by which each approach targets specific interfacial and structural factors.

### 2.1. Annealing

Annealing is a post-treatment process for thin films, in which thermal energy drives the formation of ordered microstructures in semiconductor materials [20]. Heating the coated semiconductor material above its glass transition temperature, followed by gradual cooling, facilitates molecular self-assembly and the reorganization of molecular chains, thereby enhancing crystallization and promoting the formation of more ordered crystal domains. The process supplies adequate kinetic energy and time for molecular mobility, allowing the molecules to spontaneously adopt a stable configuration characterized by lower energy and higher structural order [21]. This directly reduces the number of grain boundaries—the main barriers to charge transport—and smooths grain-boundary connections through molecular rearrangement, thereby lowering the probability of charge scattering or trapping at grain boundaries and improving signal stability. Accordingly, numerous performance-enhancement techniques incorporate annealing, as it not only releases residual energy but also establishes a stable, high-performance interface.

Accurate temperature selection significantly affects the performance of OSCs. Investigations into the annealing temperature dependence of TTT-C14 films revealed that annealing at 70 °C enhanced molecular ordering by promoting side-chain segment mobility. This treatment resulted in a 35% increase in the size of the TTT-C14 crystallites, raised the edge-on orientation ratio to 99%, enlarged grain size while decreasing grain boundaries, and generated a highly coherent charge-transport channel. Consequently, these microstructural optimizations reduced crystal-boundary scattering and trap capture during charge transport, increasing μ to 3.3 × 10^−2^ cm^2^/V·s and shifting Vth toward −6 V. Annealing also effectively decreased the trap-state density within both the semiconductor and its interface. However, the process has an optimal temperature range. Annealing at 150 °C caused side-chain melting, disrupting the original ordered stacking. As a result, μ dropped to 4.4 × 10^−3^ cm^2^/V·s, Vth shifted rightward by 6 V, and the SS widened due to increased defect density. Additionally, the I_on_/I_off_ ratio decreased to 10^3^ due to elevated leakage current [22].

**Figure 2:**
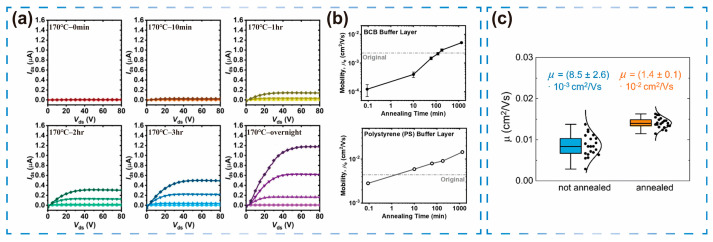
Comparison of electrical properties before and after annealing. (**a**) Comparison of OFET mobility values obtained using as-cast films versus films subjected to annealing. (**b**) Corresponding charge mobility trends over time for devices incorporating BCB buffer layer and polystyrene (PS). Reproduced with permission from [23], copyright from © The Royal Society of Chemistry 2024. (**c**) Comparison of mobility between annealed and non-annealed materials. Reproduced with permission from [24], copyright from © 2025 American Chemical Society.

Sabury et al. [23] reported that an organic film annealed at 250 °C in the liquid-crystal phase, followed by gradual cooling to ambient temperature, developed a highly ordered crystalline structure with long-range order. During heating, non-covalent conformational bonds dissociated and chain segments reoriented. Subsequently, during cooling, these bonds reformed, stabilizing a more planar and more conjugated molecular conformation. This structural reorganization enhanced μ from 8.5 × 10^−3^ cm^2^/V·s to 1.4 × 10^−2^ cm^2^/V·s (Figure 2a,b).

The value of annealing extends beyond crystalline optimization during film formation to the reversal of device aging. For example, in poly(benzimidazobenzophenanthroline) (PBBL) polymer OFETs [24], prolonged air exposure resulted in oxygen and moisture adsorption at the semiconductor/dielectric interface, forming charge traps and degrading performance. Following thermal annealing at 170 °C in a nitrogen atmosphere, the adsorbed oxygen and water molecules desorbed, eliminating these charge traps and reducing interfacial charge-transport barriers and scattering. As a result, μ recovered from 1.2 × 10^−4^ cm^2^/V·s to 5.1 × 10^−3^ cm^2^/V·s (Figure 2c), while Vth decreased from approximately 50 V to 20 V. Furthermore, the I_on_/I_off_ ratio recovered from 10^2^ to 10^4^, thereby achieving a complete restoration of the aged device’s performance. This outcome confirms thermal annealing as an effective reversible repair strategy for improving the long-term stability of organic electronic devices. This interface-repair capability enables OFET sensors to maintain long-term stability in complex environments, reducing the need for calibration.

In summary, annealing leads to a more ordered molecular arrangement, resulting in a smoother and more uniform charge-transport pathway. The increased order narrows the density of states (DOS) distribution in semiconductors, reduces energy disorder during carrier transport, and enhances carrier-hopping efficiency.

### 2.2. Solvent Engineering

The primary benefit of solvent engineering lies in its ability to predetermine the microscopic morphology of the film, thereby simultaneously enhancing the electrical performance of OFETs. By modulating solvent properties and optimizing processing techniques, molecular stacking order, orientation transitions, and microstructural development can be precisely controlled. This manipulation substantially improves carrier mobility, interface quality, and the active layer’s specific surface area, leading to enhanced sensor sensitivity, selectivity, and stability.

**Figure 3:**
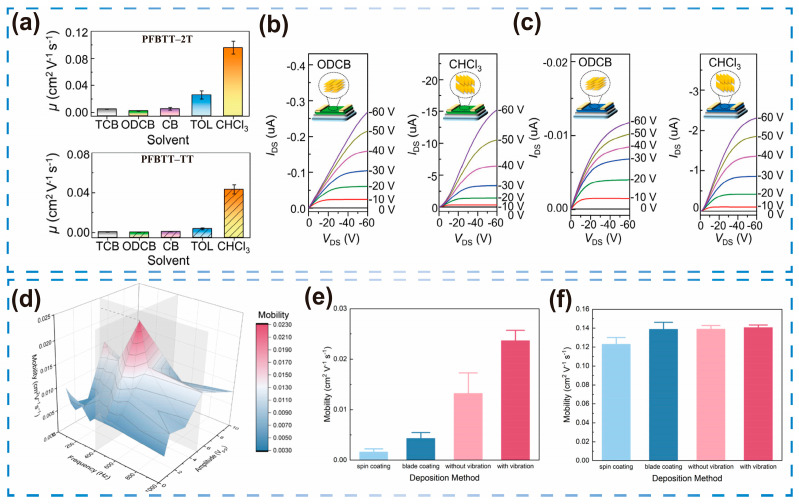
The influence of solvent selection and processing methods on OFETs. (**a**) Five solvents effect on mobility of OFET. (**b**) Output curves for PFBTT-2T and (**c**) PFBTT-TT films with ODCB and CHCl3. Reproduced with permission from [25], copyright from © 2024 American Chemical Society. (**d**) Impact of solution preprocessing and deposition methodology on P3HT film performance. (**e**) Comparison of mobility of unprocessed OFETs under different deposition methods. (**f**) Comparison of mobility of the pre-treated OFET under different deposition methods. Reproduced with permission from [27], copyright from © 2025 American Chemical Society.

Luo et al. [25] compared good solvents (1,2,4-trichlorobenzene (TCB) and 1,2-dichlorobenzene (ODCB)) with poor solvents (toluene (TOL) and chloroform (CHCl_3_)), revealing that PFBTT-2T and PFBTT-TT exhibited pronounced inter-chain aggregation in CHCl_3_. The poor solvent CHCl_3_ promoted pre-aggregation of PFBTT-2T in solution, which subsequently self-assembled during film formation into a highly ordered structure with π–π stacking parallel to the substrate in an edge-on orientation. As a result, μ increased from 5.8 × 10^−3^ cm^2^/V·s in good solvents to 1.3 × 10^−1^ cm^2^/V·s (Figure 3a).

This organized structure not only facilitates charge transport but also increases the number of active sites available for gas adsorption, thereby improving response time and the signal-to-noise ratio. Similarly, Wu et al. [26] demonstrated a transition in molecular stacking orientation from edge-on to face-on within the semiconductor layer by using the poor solvent tert-butyl bromoacetate (TBB) in conjunction with low-temperature annealing. This treatment reduced the π–π stacking distance from 3.67 Å to 3.52 Å, promoting the formation of larger and more regular aggregates. Consequently, both μ and gas adsorption efficiency improved, allowing the device to achieve a LOD of 50 ppb for NO_2_ at an ultra-low operating voltage of −0.0005 V.

While solvent engineering plays a significant role in regulating polymer aggregation, optimizing the parameters of solution processing is equally critical for achieving a favorable molecular arrangement and high-quality film formation. This is particularly evident in traditional polymers such as poly(3-hexylthiophene) (P3HT). Sun et al. [27] modified only the deposition methods, encompassing spin coating, blade coating, vibration-assisted convective deposition, and convective deposition without vibration. Among these, vibration-assisted convective deposition yielded the highest μ of 2.3 × 10^−2^ cm^2^/V·s, approximately 15 times higher than that of spin coating (Figure 3d,e). After ultrasonic treatment and aging of the P3HT solution, all four deposition methods exhibited mobility transitions (Figure 3f). Both scraping coating and convective deposition achieved μ values of 0.14 cm^2^/V·s, confirming the effectiveness of the experimental approach. Although vibration-assisted convective deposition exhibited a marginal decline to 0.12 cm^2^/V·s, performance remained high. The study highlights that a holistic approach, combining solution pre-treatment with optimized deposition, is essential for fabricating high-performance OFETs—a crucial consideration for developing reliable, stable sensors.

When transitioning from a single-polymer system to a polymer blend, a binary solvent approach becomes key to inducing controlled phase separation and facilitating microstructure development. Jiang et al. [28] introduced a binary mixed-solvent method combining two solvents with distinct volatility and solubility in a precise ratio. During solution shearing, the PDVT-10/polystyrene-block-poly(ethylene-ran-butylene)-block-polystyrene (SEBS) blend underwent controlled phase separation and self-assembly, forming continuous nanoneedle-like microstructures and ordered architectures from the nanometer to micrometer scale. Specifically, o-xylene, with its rapid evaporation rate and moderate solubility for PDVT-10, initiated polymer aggregation into core structures, while o-dichlorobenzene, with its slower evaporation rate and slightly higher solubility for PDVT-10, prolonged solute migration and promoted directional growth. Consequently, μ increased from 1.17 cm^2^/V·s in the single-solvent system to 2.71 cm^2^/V·s, while maintaining an I_on_/I_off_ ratio exceeding 10^6^. The large specific surface area provided abundant contact and diffusion channels for gas molecules, resulting in a response sensitivity to NO_2_ of 1.4 × 10^6^% ppm^−1^. Within 3 min, its response to 50 ppm NO_2_ reached 77.9 × 10^6^%, while its responses to nine interfering gases remained below 100%. However, this complex solvent system demands higher process reproducibility, a trade-off that must be considered in industrial-scale sensing applications.

After optimizing the active layer, interface engineering of the dielectric layer serves as a complementary strategy to attain both low power consumption and stable mechanical flexibility. In particular, a binary solvent system comprising t-butyl alcohol and acetonitrile was applied for solvent-engineering treatment of the polymethyl methacrylate (PMMA) dielectric layer, in conjunction with low-temperature annealing at 60 °C. This approach effectively modulated polymer chain conformation and interfacial polarity, substantially enhancing device performance, as evidenced by a low Vth (−3.5 ± 0.2 V) [29]. This interface optimization provides a more stable foundation for the sensing signal.

In conclusion, solvent engineering improves film coverage and reduces macroscopic defects such as pinholes, which act as traps. At the same time, larger grains and fewer grain boundaries further lower trap density. Similar to annealing, enhanced molecular ordering narrows the DOS distribution and improves charge transport.

### 2.3. Self-Assembled Monolayers

SAMs are molecular films one molecule thick that organize into ordered structures on various substrate surfaces. In polymer OFETs, surface modification of the dielectric layer with SAMs is primarily used to enhance the morphology and performance of the semiconductor layer.

**Figure 4:**
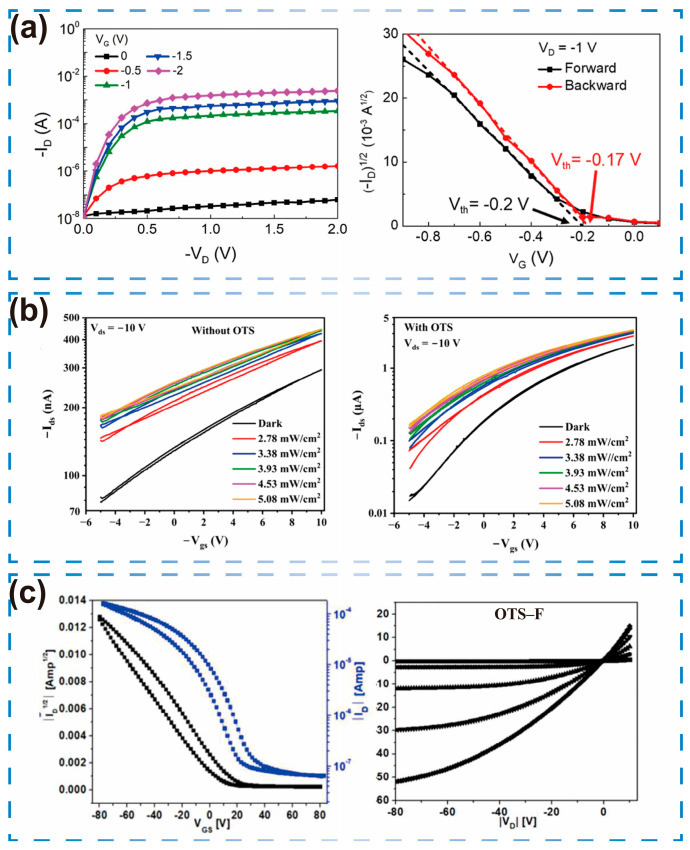
Electrical properties of different SAM. (**a**) Electrical characteristics of the P3HT-COOH OFET. Reproduced with permission from [30], copyright from © 2025 American Chemical Society. (**b**) Comparison of electrical properties under OTS and without OTS. Reproduced with permission from [31], copyright from © 2024 American Chemical Society. (**c**) Transfer and output characteristics of P3HT OFETs fabricated with OTS-F (the best). Reproduced with permission from [32], copyright from © 2025 MDPI.

Ordering at the dielectric interface not only governs the molecular arrangement of the semiconductor but also decreases the density of interface traps, thereby improving charge-carrier mobility. Lin et al. [30] investigated a polymer-based polar SAM—poly[3-(6-carboxyhexyl)thiophene-2,5-diyl] (P3HT-COOH)—as the dielectric layer, with P3HT as the semiconductor layer. The P3HT-COOH molecules spontaneously organized into an ordered monolayer, whose structured configuration and intrinsic polar field promoted the alignment of P3HT chains within the active channel. This alignment enhanced crystallinity and reduced defect density. Consequently, μ increased to 7.21 × 10^−2^ cm^2^/V·s, Vth decreased to −0.2 V, the I_on_/I_off_ ratio reached 10^4^, and SS improved to 113 mV/dec (Figure 4a).

To achieve the desired combination of low power consumption and high stability, Mandal et al. [31] chemically passivated the SiO_2_ gate-dielectric surface using an octadecyltrichlorosilane (OTS) SAM. This reduced the trap density at the semiconductor/dielectric interface from 4.5 × 10^17^ cm^−3^ to 5.7 × 10^16^ cm^−3^, improving electrical performance (Figure 4b) and enhancing the capture–release efficiency of photogenerated charges. In another study [32], optimization of the OTS SAM interface oriented the RR-P3HT edge upwards, eliminating surface traps. This enhancement proved instrumental in achieving a saturation mobility more than 150 times higher, reaching 0.18 cm^2^/V·s (Figure 4c). Furthermore, the optimization reduced Vth to 4 V, increased gain by approximately 30 times, and narrowed SS to 0.8 V/dec.

Innovation in film fabrication has become a key factor in achieving efficient and sustainable OFETs, particularly for large-scale applications. To address material wastage and poor film uniformity in conventional fabrication techniques, Baranwal et al. [33] modified the SiO_2_ gate-dielectric layer with OTS for 30 min, creating a dense hydrophobic interface. Subsequently, they employed the ribbon-shaped floating-film transfer method (FTM) to promote self-assembly and molecular orientation of polymer chains, resulting in highly crystalline and uniform films. This process enhanced μ to 0.9023 cm^2^/V·s while maintaining Vth at −1.77 V. The approach not only reduced material waste but also significantly improved the sensitivity, selectivity, and stability of the OFET ammonia-gas sensor. Even at 80 ppm NH_3_, it maintained a mobility of 0.519 cm^2^/V·s.

Similarly, Ramer et al. [34] demonstrated that using the unidirectional floating-film transfer method (UFTM) to prepare highly oriented poly [2,5-bis (3-tetradecylthiophen-2-yl) thieno [3,2-b] thiophene] (PBTTT-C14) films, in conjunction with OTES SAM modification of SiO_2_ dielectric surfaces, enhanced edge orientation and π–π stacking of molecular chains. The μ increased from 0.026 cm^2^/V·s to 0.132 cm^2^/V·s, the I_on_/I_off_ ratio rose from 1.45 × 10^4^ to 2.6 × 10^5^, and photoresponsivity improved by approximately 300 times.

Beyond dielectric-layer modification, SAMs also play a crucial role at the electrode/semiconductor interface. Luukkonen et al. [35] optimized the morphology of IDT-BT films by annealing in conjunction with a 2,3,4,5,6-pentafluorothiophenol (PFBT) SAM. This approach effectively reduced the contact resistance (RC) between the semiconductor and the gold electrode, allowing the electrolyte-gated OFET to achieve an effective μ of approximately 1–4 × 10^−3^ cm^2^/V·s at operating voltages below 1 V. Furthermore, by subjecting the samples to a soaking treatment, a modification layer formed on the electrode surface, enhancing electrical contact between the electrode and semiconductor. This treatment mitigated the impact of environmental factors on device performance and improved the operational stability of the OFET.

### 2.4. Buffer-Layer Insertion

The discrepancy in energy levels between the electrode and the OSCs gives rise to Schottky barriers, which affect charge-injection efficiency and consequently increase the RC. This interfacial mismatch plays a critical role in determining device performance, particularly under high-capacitance conditions, and may reduce the charge-carrier mobility of the device [36]. A widely adopted method to address this issue is the insertion of an insulating buffer layer at the electrode/semiconductor interface. This layer physically isolates interfacial defects and improves the uniformity of polymer film deposition [37].

It is important to note that the passivation effect of the buffer layer on interfacial defects must be coordinated with the surface properties of the dielectric layer. The dielectric surface strongly influences molecular assembly during the initial stages of semiconductor growth, thereby determining channel charge-transport efficiency. Furthermore, under an applied electric field, ion migration within the dielectric layer can increase leakage current. This elevated current not only reduces the sensitivity of OFET sensors but also induces hysteresis, ultimately compromising device stability and reliability. Wang et al. [38] addressed this issue by introducing a c-DPHA passivation layer on the surface of the polyelectrolyte dielectric, effectively inhibiting ion migration.

The incorporation of transition-metal oxide layers or poly(3,4-ethylenedioxythiophene): poly(styrene sulfonate) (PEDOT:PSS) at the electrode/semiconductor interface has been shown to effectively lower the hole-injection barrier, reduce RC, and improve the I_on_/I_off_ ratio. Wu et al. [39] deposited a PEDOT:PSS buffer layer onto the semiconductor surface, mitigating the energy-level mismatch between the electrode and the semiconductor. This modification reduced the Schottky barrier at the Ag/semiconductor interface, enhancing carrier injection and charge-transfer efficiency. As a result, μ increased from 4.33 cm^2^/V·s to 10.5 cm^2^/V·s, while RC decreased significantly from 1.81 × 10^4^ to 789 Ω·cm. Furthermore, the device exhibited superior electrical and mechanical stability compared with conventional evaporated silver electrodes.

### 2.5. Dielectric-Layer Engineering

Dielectric-layer engineering enables multi-scale modulation of the physical and chemical characteristics at the interface and has emerged as a pivotal approach to overcoming the performance limitations of OFETs. Optimization strategies primarily focus on four key aspects.

First, increasing the capacitance density to achieve low-voltage operation and strong gate control is essential. High-dielectric-constant (k) materials are preferred, as a higher capacitance can induce more carriers at low gate voltages, thereby reducing operating voltage and enhancing gate control over the channel. Notably, the interface between the high-k dielectric and the semiconductor generates a higher density of induced charges. A smoother interfacial surface mitigates scattering and trap-related phenomena, facilitating improved carrier transport [40]. To tune the dielectric properties of polymer gate dielectrics, Jang et al. [41] introduced different functional groups on the Pa-E surface using a copper-catalyzed azide–alkyne click reaction, thereby regulating the dielectric constant, which increased nearly 2-fold. When the dielectric constant is dynamically adjustable, the k value can be actively tuned for varying analyte concentrations, allowing the sensor to operate within its most sensitive range.

However, merely pursuing a high-k value may have adverse effects. A more effective approach is to equivalently increase capacitance through physical structuring. In a related study, Tavasli et al. [42] incorporated high-k zirconium dioxide (ZrO_2_) metal oxide ceramic nanoparticles (NPs) into two distinct polymer matrices—poly(vinylidene fluoride-co-hexafluoropropylene) (PVDF-HFP) and cyanoethyl cellulose (CEC)—to fabricate dielectric layers. The areal capacitance increased with NP concentration, leading to greater charge accumulation at the semiconductor/dielectric interface and enabling device operation at reduced voltages.

**Figure 5:**
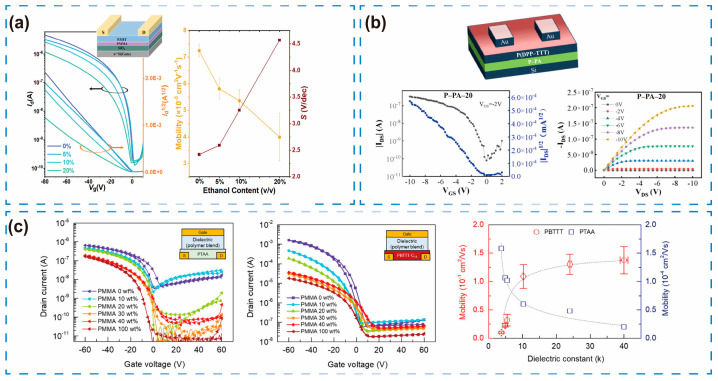
The influence of the dielectric layer on the electrical properties of OFETs. (**a**) Electrical properties of PMMA dielectric materials under different ethanol concentrations. Reproduced with permission from [44] copyright from © 1999–2025 John Wiley & Sons, Inc. or related companies. (**b**) Schematic of OFET structure and electrical characteristics of P-PA-X device. Reproduced with permission from [43], copyright from © 2025 American Chemical Society. (**c**) The transfer characteristic curves and mobility of PTAA and PBTTT-C14 semiconductors as a function of k. Reproduced with permission from [48], copyright from © 1999–2025 John Wiley & Sons, Inc. or related companies.

Phosphocholine-based polyampholyte (P-PA) polymers also exhibit high-k values that effectively reduce the contact potential difference at the interface. The optimized device, P-PA-20, achieved a μ of 1.02 cm^2^/V·s, an exceptionally low Vth of 0.07 V, and an I_on_/I_off_ ratio ranging from 10^3^ to 10^4^, all at a modest operating voltage of −2 V (Figure 5b). Compared with unmodified polyurethane (PU) devices, the P-PA-20 device exhibited a 400-fold mobility enhancement and a 60-fold reduction in Vth [43].

Second, interface trap states must be minimized to enhance carrier transport efficiency. These traps mainly originate from chemical groups, surface roughness, and dipole disorder. Although a high-k dielectric increases capacitance, it may also introduce strong dipoles that intensify trapping. The DOS width can be modulated by tailoring the dielectric’s molecular conformation. Optimization typically involves physical smoothing, such as introducing a PMMA layer—as demonstrated by Tavasli et al. [42]—to improve dielectric surface morphology and reduce leakage current. Another approach is chemical passivation and energy-level regulation. Designing the molecular conformation of the dielectric layer enables more precise energy-level control. Yang et al. [44] introduced a strategy involving the modulation of local chain conformations on the dielectric surface to fine-tune the DOS width. Specifically, a PMMA dielectric layer fabricated with pure cyclohexanone yielded the narrowest DOS (0.401 eV), corresponding to a μ of 7.24 × 10^−3^ cm^2^/V·s, an I_on_/I_off_ ratio exceeding 10^4^, and a Vth between −4 and −0.5 V (Figure 5a). The dielectric conformation affects the arrangement of interface dipoles, thereby modulating the semiconductor band-tail distribution. A narrower DOS indicates shallower trap energy levels, reduced carrier transition barriers, and increased mobility [45]. This strategy is applicable to other polymer dielectrics (such as PS, PVP), whose conformation can be optimized through solvent engineering or annealing.

Park et al. [46] systematically optimized device performance using PTCDI-C8 as the n-type semiconductor by adjusting the molar ratio of 6FDA to DOCDA in the photopatternable soluble copolyimide (ScoPI-#). When ScoPI-0 (without 6FDA) was used as the gate dielectric, the device exhibited optimal electron mobility (0.023 cm^2^/V·s), a positively shifted Vth, reduced SS, and an increased on–off ratio. These improvements were mainly attributed to the high-k dielectric provided by the DOCDA segment, which enhanced electron accumulation in the channel, while its lower surface energy and fewer interface traps reduced carrier scattering and capture, improving interface quality. Although fluorine atoms introduced by 6FDA could lower surface energy and passivate interfacial defects, their dipole effect weakened electron accumulation in the n-channel and inhibited device performance. Therefore, a high 6FDA content should be avoided in n-type devices.

Third, controlling semiconductor orientation and crystallinity via surface topological or chemical induction from the dielectric layer improves charge-transport pathways. Bond et al. [47] introduced an innovative approach using expanded-poly(tetrafluoroethylene) (ePTFE) micro-membranes—characterized by high specific surface area, flexibility, and chemical inertness—as gate dielectrics, in place of conventional SiO_2_. Annealing at 200 °C caused thermal contraction of the ePTFE fibers, reducing the effective gate–semiconductor distance and enhancing the equivalent capacitance density. This thermal shrinkage not only shortened the effective dielectric thickness and increased capacitance but also induced the orientation of semiconductor molecules through the micro-fiber topology, reducing π–π stacking disorder and DOS discreteness. The PTFE lattice further promoted preferential alignment of PDPP4T chain segments, resulting in more ordered π–π stacking. Consequently, the μ of the ePTFE-2 device reached 1.8 cm^2^/V·s, representing a twentyfold increase over the SiO_2_ control (μ = 9 × 10^−2^ cm^2^/V·s).

Finally, achieving a balance between dielectric constant and dipole interactions is essential to prevent carrier localization from excessively strong dipolar effects. This interplay is reflected in the relationship between k and μ, commonly referred to as the k–μ correlation. Jeong et al. [48] reported that in PBTTT-C14, a semi-crystalline polymer, side chains increased the separation between the semiconductor and dielectric layers, substantially reducing dipole interactions. In contrast, PTAA, an amorphous polymer, exhibited a shorter polymer–dielectric distance, resulting in stronger dipole interactions and a negative k–μ correlation (Figure 5c).

Fluctuations in the dipole electric field modulate the semiconductor band-tail states and increase carrier scattering. Thus, isolating the high-k material from the semiconductor through interface engineering is essential. A double-layer or composite dielectric structure can be employed to achieve a high dielectric constant [49,50]. For instance, the PMMA/PVA double-layer dielectric configuration achieved a μ of 7.8 × 10^−2^ cm^2^/V·s, an I_on_/I_off_ ratio of 2.8 × 10^6^, a Vth near 1.3 V, and an SS of 0.15 V/dec at operating voltages below 5 V [51]. The PMMA layer suppressed interfacial dipole fluctuations and reduced remote phonon scattering at the surface, substantially improving the overall electrical performance and photostability of low-voltage n-type OFETs. Similarly, combining low-k PS with high-k poly(vinylidene fluoride-trifluoroethylene) [P(VDF-TrFE)] increased n-type OFET μ from 0.02 to 0.07 cm^2^/V·s and shifted Vth toward 0 V. The inert PS surface reduced chemical traps and SS, while the polar P(VDF-TrFE) groups optimized charge injection through energy-level alignment and reduced RC [52]. Furthermore, the Cytop/parylene C double-layer dielectric effectively encapsulated the semiconductor layer, restricting water and oxygen ingress [53], making it suitable for sensing applications in complex environments. The parylene/PVA double-layer dielectric also effectively suppressed leakage current, yielding a μ of 2.9 × 10^−2^ cm^2^/V·s and an I_on_/I_off_ ratio of 10^3^ [54].

In addition, a double-layer dielectric composed of thermoplastic polyurethane (TPU) and an imidazolium-based ionic gel ([EMIM][BF4]) achieved a high areal capacitance of 1.27 μF/cm^2^ and a low leakage current below 10^−6^ A [55]. Studies on anion size effects within [EMIM][BF4] revealed that the [EMIM][BF4]/TPU system, with the smallest anion, exhibited both high capacitance and rapid formation of dense electric double layers (EDLs) at the gate and semiconductor interfaces. This resulted in an exceptionally high carrier density at low operating voltages while maintaining high capacitance and significantly suppressing leakage current.

Beyond double-layer dielectrics, Zhang et al. [56] enhanced stretchable OFET performance by developing a three-layer elastomer dielectric composed of NBR/SEBS/NBR-C. The high-k NBR boosted capacitance density and enabled sufficient carrier accumulation at low voltages. SEBS served as a buffer to minimize energy-level mismatches caused by high-k polymer polarization, reducing interface trap density. NBR-C provided solvent resistance and mechanical stability. This three-layer design improved charge injection and transport by increasing capacitance density and lowering interface trap states, which decreased SS and enhanced the device’s switching behavior and response speed. The device achieved a high μ of 5.7 × 10^−2^ cm^2^/V·s (up from 2.01 cm^2^/V·s) at a low operating voltage (−5 V), an I_on_/I_off_ ratio exceeding 10^5^, a low Vth (−0.1 V), and excellent mechanical stability, maintaining nearly constant mobility even under 100% strain.

### 2.6. Main-Chain Engineering

**Figure 6:**
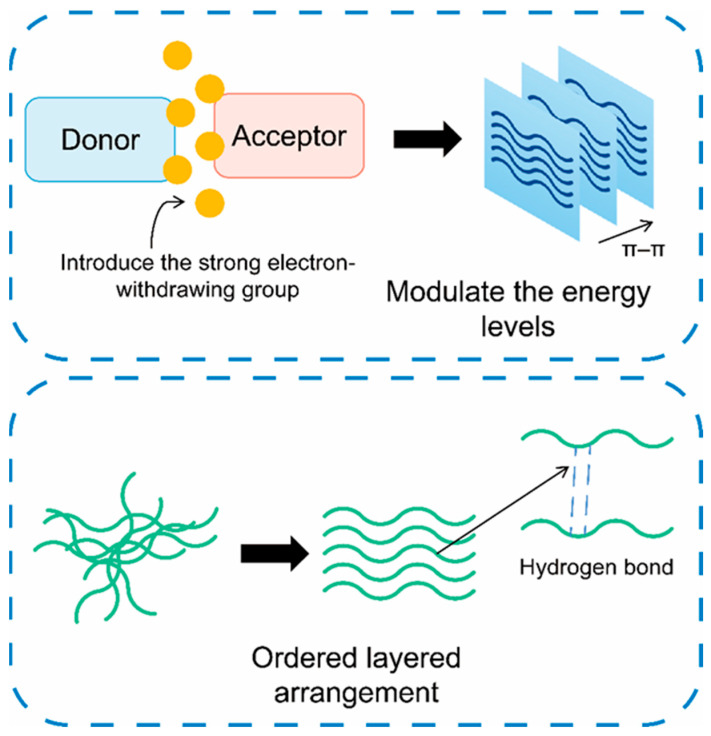
Schematic diagram of the main-chain engineering strategy.

Main-chain engineering directly regulates the intrinsic properties of polymer semiconductors through chemical structure innovations. This is achieved through various methods, ranging from inducing self-assembled hydrogen bonds to precisely designing the acceptor unit’s energy level, thereby achieving synergistic improvements in both charge transport and stability (Figure 6).

In a seminal study, Yu et al. [57] introduced latent hydrogen bonds (t-Boc) into the main chain of the QA unit, using this polymer as the semiconductor layer in OFETs. Upon thermal annealing at 180 °C, non-covalent bonds (hydrogen bonds) were induced, triggering molecular self-assembly. During annealing, the t-Boc groups dissociated, exposing highly polar amide bonds that formed dense networks of intermolecular hydrogen bonds between adjacent polymer chains. This dynamic bonding process triggered the self-assembly of the molecules, driving the polymer chains to transform from a disordered to a highly ordered layered arrangement. The resulting phase transition enhanced π–π stacking, shortened the inter-chain distance, and substantially improved the efficiency and path continuity of carrier hopping transmission, thereby increasing μ to 1.02 cm^2^/V·s, with Vth = −17 V and an I_on_/I_off_ ratio of 10^5^–10^6^.

For n-type polymers, the key optimization challenge lies in achieving a low and stable LUMO energy level to enable efficient electron injection and environmental stability. To address the bottleneck wherein electron mobility in n-type polymers remains much lower than that in p-type materials, the electronic defect strength and steric hindrance of the main-chain receptor units have become critical factors governing electron injection efficiency. In one study [58], the transformation from p- to n-type charge transport was achieved by introducing varying numbers of cyano substituents into the polymer main chain to tune the electronic structure and aggregation behavior. Furthermore, among four new diketopyrrolopyrrole (DPP)- and thienopyrrolodione (TPD)-based copolymers, the pyridine-linked P3-[Py] achieved an electron mobility of 4.6 × 10^−2^ cm^2^/V·s, which—the highest level reported to date [59].

By introducing diphenylenyl groups at the vinylene bridging sites of the TVT unit, the intrinsic contradiction between electronic structure regulation and molecular packing optimization was effectively balanced [60]. The diphenylenyl substitution not only further reduced the LUMO energy level from P1’s −3.83 eV to −3.90 eV, enhancing the electron injection efficiency, but also constructed a more alternating D–A1–D–A2 structure, significantly enhancing the intramolecular charge transfer (ICT) effect. Ultimately, the electron mobility increased from P1’s 0.52 cm^2^/V·s to P2’s 1.03 cm^2^/V·s, the Vth decreased, the subthreshold swing was optimized to 3.0–3.5 V/dec, the I_on_/I_off_ ratio remained at 10^5^–10^6^, the film roughness was only about 0.8–1.0 nm, and it had an extremely low defect density. These characteristics enable it to exhibit comprehensive advantages of high sensitivity, low power consumption, and good stability in n-type OFET sensor applications, especially suitable for sensing scenarios that require high electron mobility and low operating voltage.

A fluorinated selenophene–ethylene–selenophene (FSVS) structure was also introduced into the polymer main chain [61]. The fluorine atoms significantly lowered the LUMO energy level through inductive and conjugation effects, reducing both the injection barrier and Vth. Simultaneously, they enhanced intermolecular stacking through non-covalent interactions, shortened the π–π stacking distance to 3.7 Å, and induced a face-on to edge-on transition in film orientation, optimizing charge-transport pathways. The low-k dielectric layer CYTOP (k ≈ 2.1) further reduced interfacial polarization disorder, suppressing energy disorder and trap-state density. Consequently, the n-type OFET achieved a mobility of 0.32 cm^2^/V·s and an I_on_/I_off_ ratio exceeding 10^6^.

Notably, non-covalent locks between receptor and donor units can concomitantly enhance main-chain planarity and intermolecular interactions. Distinct receptor units interacting with donor cores through π–π stacking can substantially alter molecular mobility. As reported by Alam et al. [62], three new donor–acceptor (D-A) copolymer families—PDPP-2FTVT, PIIID-2FTVT, and PNDI-2FTVT—have been identified in relation to different receptor units. Among them, DPP formed a non-covalent lock with 2,2′-(1E)-1,2-ethenediylbis[3-fluorothiophene] (2FTVT), yielding the longest conjugated length. Its HOMO energy level (−5.52 eV) closely matched the Au electrode (approximately −5.1 eV), resulting in the lowest hole-injection barrier. Consequently, PDPP-2FTVT exhibited the most planar main chain, optimal energy alignment, preferred molecular orientation, and highest crystallinity, achieving the highest hole mobility of 1.93 cm^2^/V·s among current 2FTVT systems.

Designing double-receptor backbones enhances dipole–dipole interactions through polar groups, promoting multi-dimensional molecular ordering. Kranthiraja et al. [9] pioneered the design and evaluation of a new class of dual-acceptor CPs based on DPP and dioxo-benzodithiophene (BDD). The polar groups of the receptor units enhanced intermolecular dipole–dipole interactions, promoting ordered packing and more regular edge-on or face-on orientations. Among these, DPPF-BDD outperformed similar single-receptor polymers, exhibiting a high electron mobility of 0.0503 cm^2^/V·s after doping. The smaller chalcogen atoms (O < S < Se) in the structure reduced side-chain steric hindrance, enabling tighter stacking and higher crystallinity.

However, the conjugation length of the main chain must balance rigidity and flexibility. Excessive elongation disrupts π–π coupling, whereas moderate extension optimizes interlayer order. As shown in one study [22], precise control of molecular packing and crystal orientation can be achieved by varying the number of thiophene units in the polythiophene main chain and adjusting main-chain rigidity and side-chain density. Notably, the medium-length main chain (TT-C12) exhibited the most compact π–π stacking (0.37 nm), representing an optimal balance of rigidity and flexibility and yielding an edge-on orientation ratio of 95%. This structure significantly reduced steric hindrance, promoted side-chain intercalation, and improved interlayer order, increasing saturation-zone mobility from 6.9 × 10^−4^ cm^2^/V·s (C12) to 3.4 × 10^−2^ cm^2^/V·s—a nearly fiftyfold improvement. It also shifted Vth from −16 V to around −12 V, while the ion/current increased by two orders of magnitude (to 10^5^) due to suppressed leakage current. However, excessive main-chain elongation (TTT-C12) caused the molecular chain to tilt due to excessive rigidity and weakened π–π coupling, reducing the orientation ratio and mobility to 2.2 × 10^−2^ cm^2^/V·s.

Challenging the traditional crystallization paradigm, a near-amorphous main chain can form a three-dimensional charge-transport network through high linearity and strong coplanarity. The representative material pIDTBT, although lacking long-range order, relies on main-chain linearization to form a short-range, network-like ordered structure. Recent research introduced a previously unexplored 1,4-dihydropentalene bridging structure to design two isomeric monomers (anti-C16DHIT-Br and syn-C16DHIT-Br), which regulated the main-chain conjugation path, significantly enhancing main-chain planarity and molecular packing order, thereby achieving a hole mobility of 2.38 cm^2^/V·s [63].

### 2.7. Side-Chain Engineering

**Figure 7:**
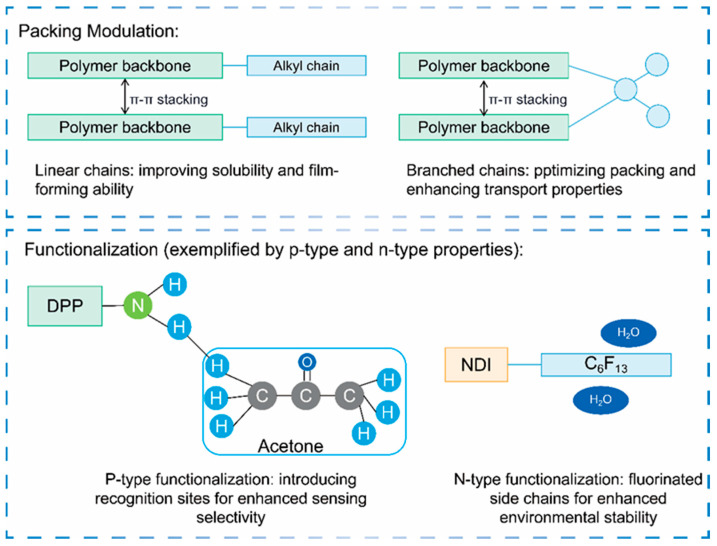
Schematic diagram of the side-chain engineering strategy.

Side-chain engineering reduces spatial steric hindrance and enhances molecular order and π–π stacking by precisely controlling alkyl-chain length, branching position, and functional-group organization (Figure 7). As a result, it significantly improves charge mobility and device stability. This mechanism has been systematically discussed in numerous studies. The latest review [64] comprehensively summarized various side-chain engineering strategies—such as alkyl-chain regulation and side-chain functionalization—that significantly enhance OFET performance, including mobility.

As a key regulatory factor, alkyl-chain length reorganizes film order through the self-plasticization effect. When the main chain is fixed, extending the side chain induces molecular rearrangement. In CPs with a TTT main-chain structure, varying the alkyl side chain length from C6 to C14 significantly optimized film structure and device performance. The longer C14 chain utilized the self-plasticization effect to enhance molecular order and promote stronger side-chain intercalation. It also increased the edge-on orientation ratio to 97% and optimized π–π stacking by reducing the distance between the main chains. This slight reduction exponentially enhanced molecular orbital overlap, increasing μ from 1.1 × 10^−2^ cm^2^/V·s for C6 to 2.3 × 10^−2^ cm^2^/V·s for C14. This brought Vth closer to −10 V and synchronously increased the I_on_/I_off_ ratio to 10^4^ [22] (Figure 2a,b).

The π–π stacking distance directly determines the degree of orbital overlap between adjacent conjugated frameworks in OSC films, thereby affecting the transition probability of charge carriers (electrons or holes) between chains. Regulating spatial steric hindrance is a more effective way of compressing the π–π stacking distance than extending the side chains. Side-chain engineering is also crucial for n-type polymers. By selecting suitable branched alkyl chains, steric hindrance can be minimized and π–π stacking compressed, significantly enhancing electron mobility. For instance, Cao et al. [65] used a rigid, nearly amorphous n-type polymer PBN27 as the platform. Within the semiconductor layer, the π–π stacking distance decreased from 3.55 Å to 3.41 Å, increasing electron mobility from 0.041 cm^2^/V·s to 0.30 cm^2^/V·s while maintaining an I_on_/I_off_ ratio of 10^5^.

Functionalization of side chains can also transform semiconductors into active sensing elements. Introducing an aniline group (-NH_2_) transforms the DPP copolymer from a passive semiconductor into an active sensing platform, as the hydrogen-bonding sites of the aniline group can specifically capture analytes. Mayarambakam et al. [66] incorporated -NH_2_ groups into a DPP–thiophene copolymer, synthesizing seven DPP-based polymers with aniline side chains. By varying the aniline substituents (H, OCH_3_, Cl) and the ratio to alkylated DPP (1:1 or 1:2), they optimized polymer solubility, film-forming ability, and charge-capture capability.

In complex application scenarios, single side-chain strategies often involve trade-offs. However, a hybrid side-chain design overcomes this issue by combining rigid and flexible components. Wang et al. [67] introduced hybrid side chains to the polymer main chain via side-chain modification, systematically regulating intermolecular aggregation and film microstructure. As a result, the hole mobility of OFETs based on the halogenated solvent chlorobenzene (CB) increased from 5.10 cm^2^/V·s to 6.13 cm^2^/V·s. Even in the non-halogenated solvent o-xylene (o-XY), the mobility was still 3.02 cm^2^/V·s, significantly improving device performance and operational stability. The molecular-level combination of linear and branched chains enhanced packing density and ensured processing adaptability.

### 2.8. Blending and Doping

**Figure 8:**
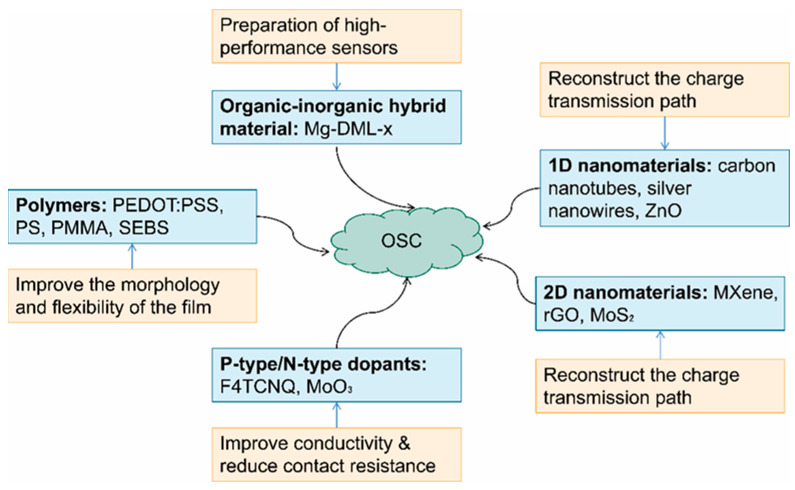
Schematic diagram of blending and doping strategies.

Incorporating additional substances into the material can optimize its properties. This may involve adjusting the phase-separation structure in blending systems or modifying the energy levels through doping strategies. The common goal is to reduce energy disorder and enhance carrier mobility. In blend systems, the polymer binder improves the film-forming properties of small molecules and plays a crucial role in charge transport. Selecting an appropriate polymer binder requires considering energy-level matching to ensure efficient charge transport. Figure 8 classifies various materials that can be blended or doped with OSCs and explains how different materials can be selected to achieve specific optimization effects.

Organic–inorganic hybridization first achieved a marked improvement in charge transport through interfacial electronic engineering. Mg-doped DML-x was blended with P3HT, where strong electronic interactions between the Lewis-basic Mg framework and NO_2_ gas increased the hole mobility (μ) from 7.06 × 10^−4^ cm^2^/V·s to 2.3 × 10^−3^ cm^2^/V·s and raised the I_on_/I_off_ ratio by two orders of magnitude to 10^4^ [68]. This interaction not only enhanced mobility but also improved gas selectivity and sensitivity.

Leveraging the high conductivity and Schottky barrier modulation of Ag NWs increased the mobility of P3HT tenfold to 4 × 10^−3^ cm^2^/V·s, while the I_on_/I_off_ ratio improved by a similar factor to approximately 10^4^ [69]. MXene nanobelts achieved a highly oriented structure through UFTM, increasing the μ of P3HT from 0.05 to 0.58 cm^2^/V·s [70].

Low-molecular-weight polymer additives can optimize both electrical performance and environmental stability through precise vertical phase separation [71]. Blending short-chain PS with OSCs, while leveraging molecular-weight-controlled vertical phase separation, resulted in the formation of a hydrophobic barrier layer at the bottom of the device. This led to a substantial improvement in electrical performance and water stability. The μ increased 25-fold to 2.5 × 10^−2^ cm^2^/V·s, Vth decreased from 15.6 V to 3.7 V, and the I_on_/I_off_ ratio rose 100-fold to approximately 10^4^.

Precise optimization of the mixing concentration is imperative. Excessive blending can cause phase-separation defects, whereas moderate adjustment can enhance electrical stability. Urbancic et al. [72] reported that incorporating a mixed film of MXene and P3HT into OFETs significantly increased P3HT carrier mobility from 1.5 × 10^−3^ cm^2^/V·s to 2.0 × 10^−3^ cm^2^/V·s. The study found that a low concentration of MXene (2.5%) reduced the RC, improving charge injection; however, higher concentrations (5%) caused MXene aggregation, increased channel resistance, and severe charge trapping, resulting in a decrease in mobility. Therefore, the doping concentration must be optimized to balance performance and stability.

Incorporating molecular dopants can further reduce energy disorder. Interactions between dopants and the semiconductor significantly alter interfacial charge distribution, thereby improving mobility [73,74]. However, excessive dopants act as charge-scattering centers, disrupting molecular order and leading to a significant decrease in the I_on_/I_off_ ratio. Therefore, the doping level must be precisely optimized to balance improved sensitivity against potential reductions in selectivity and dynamic range.

Kumar et al. [75] synthesized MoS_2_ nanosheets via ultrasonic-assisted liquid-phase exfoliation and combined them with a low-bandgap CP, PCPDTBT, to construct a PCPDTBT/MoS_2_ composite active layer. This process induced polymer chain self-assembly, enhanced π–π stacking and long-range order, and significantly improved the charge-transport performance of bottom-gate top-contact OFETs. Carrier mobility increased from 0.98 × 10^−3^ cm^2^/V·s for pristine PCPDTBT to 2.70 × 10^−3^ cm^2^/V·s. Concurrently, Vth decreased from −25.1 V to −10.1 V, the I_on_/I_off_ ratio increased from 0.95 × 10^2^ to 4.3 × 10^2^, and SS remained at approximately 11.7 V/dec.

Balancing dopant levels and aggregation order is crucial. Doping P3HT with 2,3,5,6-tetrafluoro-7,7′,8,8′-tetracyanoquinodimethane (F4TCNQ) significantly enhances electrical conductivity and broadens the light absorption spectrum, generating a broadband response [76]. Zhang et al. [77] reported that at a 1 wt.% F4TCNQ doping level, P3HT films exhibited maximum charge mobility and conductivity. The dopant not only increased the carrier concentration in the P3HT film but also altered the polymer’s aggregation state and crystallinity through interaction with the P3HT chain. However, higher doping levels hindered aggregation ordering and decreased device mobility.

To meet the mechanical stability requirements of flexible electronics, the phase-separated structure of SEBS elastomer and CPs can simultaneously optimize charge-transport pathways and stress dissipation networks. The elastomer does not merely dilute the semiconductor but forms a continuous phase to dissipate mechanical stress, while the CP creates a permeable network within it to facilitate charge transport. This structure enables high electrical performance under strain. Shi [55] used strong π–π interactions between P3HT and SEBS to promote P3HT aggregation, forming a continuous and ordered polymer network. This device retained 86% of its performance after 1000 cycles of 10% stretching, with μ decreasing only from 5.19 cm^2^/V·s to 3.60 cm^2^/V·s. Gong et al. [78] mixed P3HT and SEBS at a 1:1 ratio via spin coating, which spontaneously formed a continuous microgrid structure. The measured μ was found to be 2.5 × 10^−2^ cm^2^/V·s, with an I_on_/I_off_ ratio of 1.8 × 10^4^, an SS of approximately 1.2 V/dec, and a Vth below 1 V. Remarkably, the device maintained performance under a 50% tensile strain. Jeong et al. [79] further optimized the DPPT-TT/SEBS blend by tuning the hard/soft segment ratio in SEBS to align nanofiber morphology with electrode and dielectric stability. The resulting device achieved a high mobility of 0.5 cm^2^/V·s at 50% tensile strain, with an I_on_/I_off_ ratio of 10^5^, and a mobility retention rate of over 90% after 10,000 stretching cycles, which was far superior to the unoptimized system.

**Figure 9:**
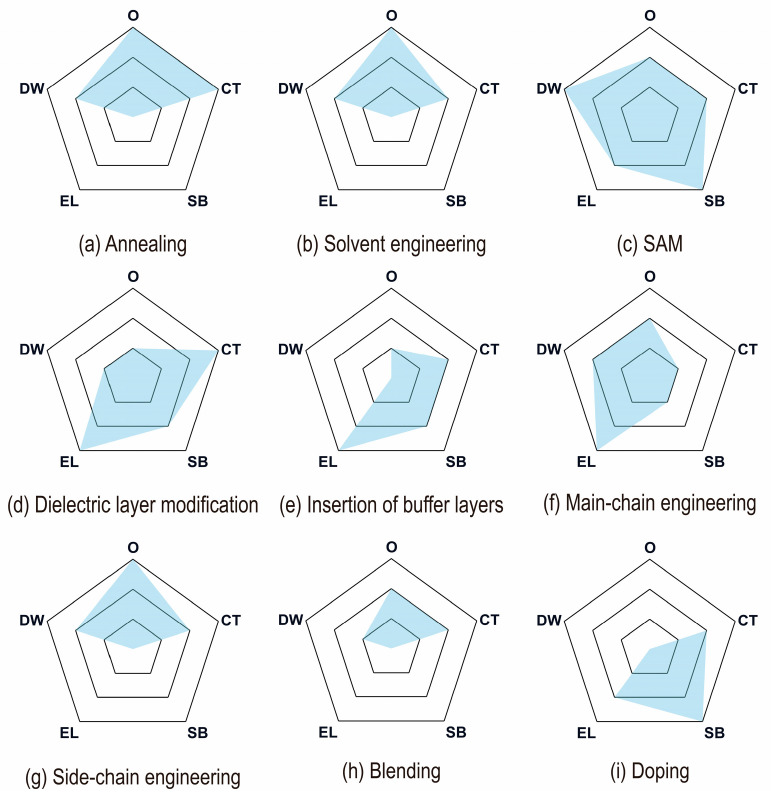
Comparison of polymer OFET optimization strategies. Nine commonly used optimization strategies, i.e., (**a**) annealing, (**b**) solvent engineering, (**c**) SAMs, (**d**) insertion of buffer layer, (**e**) dielectric layer engineering, (**f**) main-chain engineering, (**g**) side-chain engineering, (**h**) blending, and (**i**) doping, are evaluated to terms of improving the ordered structure of OSCs (O), reducing charge traps (CT), reducing Schottky barriers (SB), mitigating energy level mismatch (EL), and modulating the DOS width (DW).

To sum up, Figure 9 provides a detailed summary of these optimization strategies, qualitatively analyzing and comparing five representative improvement scenarios.

## 3. Application of Performance Optimization Strategies in Sensor Platforms

**Figure 10:**
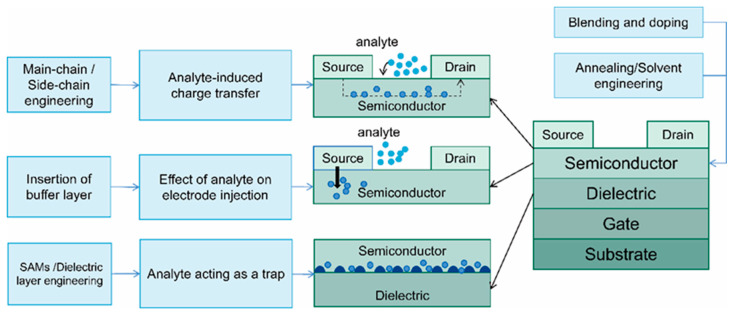
Schematic diagram of the sensing mechanism and optimization strategies of polymer OFET.

Optimizing the electrical parameters of OFETs ultimately aims to enhance their core performance as sensors. Figure 10 illustrates the relationship between the sensing mechanism of polymer OFETs and the corresponding optimization strategies. When an analyte—such as an oxidizing gas—undergoes charge transfer with the semiconductor, this process is analogous to chemical doping. Such interaction directly modifies the carrier concentration within the channel, leading to a shift in Vth and an increase in current [4,26,80]. Alternatively, when an analyte—such as a polar molecule—becomes trapped at the critical interface between the semiconductor and dielectric layer, it acts as a charge trap and hinders carrier transport. This reduces mobility and degrades switching characteristics. Furthermore, when the analyte adsorbs onto the dielectric surface, its dipole moment induces an additional electric field [81], equivalent to the application of an extra gate voltage, thereby causing a shift in Vth [82]. Collectively, these interactions fundamentally influence the primary performance metrics of the sensor. This section examines how various optimization strategies contribute to improved sensing performance in OFET-based sensor platforms.

### 3.1. Enhancing Sensitivity and Lowering Detection Limit

Sensitivity—the ability of an OFET sensor to convert minute analyte signals into measurable electrical responses—is directly limited by the device’s charge-transport efficiency. Sensitivity and LOD collectively characterize a sensor’s capacity to identify weak signals. The critical factor in this process is attaining an optimal signal-to-noise ratio, which necessitates that the sensing platform produces a sufficiently robust electrical signal—facilitated by high μ and low SS to enhance signal amplification—while concurrently ensuring that device noise remains minimal, achievable through a high I_on_/I_off_ ratio and stable Vth to effectively suppress noise [69]. Therefore, optimizing the charge-transport pathway by increasing carrier mobility is the fundamental approach to enhancing sensitivity. Section 2 described several strategies for enhancing carrier mobility.

On the one hand, interface and morphology engineering are systematically integrated to enhance both transport and adsorption processes [83]. The objective is to regulate the physical structure while simultaneously improving charge transport efficiency and analyte accessibility.

Solvent engineering serves as a fundamental strategy for optimizing the morphology of thin films. For example, Jiang et al. [28] proposed a binary solvent strategy that achieved high sensitivity, selectivity, and stability in wearable gas sensing applications. Through the modulation of crystallization kinetics, they successfully fabricated semiconductor films characterized by a highly ordered and crack-free morphology. This structural refinement not only improved the charge carrier mobility and an I_on_/I_off_ ratio of the devices, thereby establishing a highly conductive baseline conducive to signal amplification, but also ensured consistent sensing responses attributable to the uniform microstructure.

In another study [84], reduced graphene oxide (rGO) was incorporated into a PEDOT:PSS composite system. The π–π stacking and phase separation between rGO and PEDOT chains boosted carrier mobility from 0.00549 cm^2^/V·s to 0.02 cm^2^/V·s (+264%) without any post-treatment. This mechanism provided a highly conductive baseline and low noise floor, amplifying the current attenuation signal (ΔI) induced by Hg^2+^ binding. The device exhibited a pronounced current attenuation within 2–3 s for Hg^2+^ concentrations from 1 to 60 nM, with a LOD as low as 2.4 nM. These results demonstrate that enhancing μ directly amplifies the signal baseline, which is crucial for detecting ultra-low analyte concentrations.

A sensing-function-oriented blend system can sacrifice certain electrical properties to achieve an extremely high response rate. Fundamentally, it involves accepting a marginal reduction in mobility in return for an enhancement in analyte capture efficiency or diffusion rate by an order of magnitude. Hong et al. [85] uniformly incorporated UiO-66 into the P3HT semiconductor layer, constructing high-density NO_2_ adsorption sites within the semiconductor and at the semiconductor–environment interface. Despite a slight decrease in mobility to 0.002 cm^2^/V·s, the OFET’s gas-sensing performance was doubled, achieving highly selective NO_2_ detection. Blending proves to be an effective strategy for attaining high sensitivity when the augmentation in the density of active sites adequately offsets the transmission loss.

A separate investigation [86] successfully fabricated uniform nanopores into an 8 nm ultrathin film by blending P3HT with PS. This unique structure not only optimized polymer chain alignment and crystallinity, boosting charge mobility by 41%, but also increased the specific surface area 19-fold, thereby substantially enhancing the adsorption and diffusion of NO. Consequently, the sensor’s NO response increased from 28% to 42%, sensitivity improved from 3.1 to 4.7% ppm^−1^, and the LOD decreased to 0.5 ppm. These findings underscore the efficacy of the blended configuration in synergistically augmenting both transport properties and adsorption capacity. Similarly, a P3HT composite film containing 10 wt.% g-C_3_N_4_ exhibited approximately twice the charge mobility of pure P3HT [80]. This blend performed optimally when detecting 10 ppm NO, yielding a response rate of 40.6%, response time of 129 s, recovery time of 148 s, and excellent repeatability over seven cycles. These results were attributed to the large surface area of g-C_3_N_4_, which provided abundant active sites, enhanced charge transfer with P3HT, and increased carrier mobility.

On the other hand, intrinsic performance can be improved through molecular engineering. By strategically designing molecular structures, it is possible to fundamentally modulate the electronic properties and aggregation behavior of the materials.

Main-chain engineering regulates the intrinsic electronic structure and aggregation behavior of polymers through targeted chemical synthesis. In a study on H_2_S detection [87], a PCPDTBT-based OFET sensor achieved a LOD below 1 ppm and a high response of 71.3% at 1 ppm H_2_S. Sulfur atoms within the polymer backbone engaged in strong van der Waals interactions with the lone-pair electrons of H_2_S, promoting selective adsorption. Furthermore, the FTM and solvent vapor annealing (SVA) were used to optimize film morphology, reducing grain size and increasing the number of grain boundaries. These changes not only improved charge-transport efficiency but also created more active sites for gas adsorption, further enhancing the sensing signal.

In contrast, side-chain engineering optimizes performance without altering the conjugated main-chain structure through precise spatial and electronic modulation. Mayarambakam et al. [66] demonstrated that side-chain functionalization could enhance device performance. Their approach produced a current response of approximately 5.8% to 50 ppm acetone gas—about twice that of non-functionalized PDPP4T—and achieved an even higher response of 30–60% in a solution environment, highlighting the effectiveness of molecular engineering in boosting sensitivity.

In summary, whether through post-treatment (such as solvent engineering), molecular engineering, or composite formation, the core objective is to achieve efficient charge transport, thereby converting weak chemical interactions into pronounced electrical signals. This, in turn, enhances sensitivity and lowers the LOD.

### 3.2. Improving Selectivity and Specificity

Selectivity—the ability to distinguish a target analyte from interfering substances—is critical for sensor applications in complex environments. In OFET sensors, high selectivity is primarily achieved by constructing specific recognition interfaces or leveraging unique chemical interactions.

The work by Meresa et al. [88] is a prime example of using doping to enhance selectivity. Although p-doping with TPFB slightly reduced the field-effect mobility of P3HT, it significantly increased the initial on-current. More importantly, the strong affinity between TPFB and NH_3_ molecules enabled a selective sensing mechanism. The device response to NH_3_ surged from 20.3% to 65.8% at 100 ppm, while responses to interfering volatile Organic Compounds such as acetone, methanol, and dichloromethane remained minimal (0.7–2.1%). This example clearly illustrates how an appropriate dopant can impart high specificity, even when fundamental electrical parameters like mobility are slightly compromised.

Interface engineering has also become a research hotspot. One study [89] constructed an extended-gate-type OFET immunosensor by integrating the high-mobility p-type polymer semiconductor, PBTTT, with a C14-PA/AlOx self-assembled dielectric layer and introducing CPT-SAM on the gate surface for targeted immobilization of anti-CgA antibodies. This configuration enabled highly sensitive, label-free detection of glycoprotein CgA. The self-assembled dielectric layer not only increased the dielectric capacitance to approximately 0.8 μF/cm^2^—significantly enhancing the gate’s electrostatic control over channel charges—but also reduced interface trap density and leakage current, allowing stable operation at voltages below <3 V. Consequently, small charge variations caused by protein binding were effectively amplified into significant threshold-voltage shifts. The device achieved a LOD as low as 0.11 μg/mL, reducing the detection time by 30 min compared with the 2.5-h traditional ELISA method, while maintaining a response of less than 5% to interfering proteins such as IgA and amylase.

Zhang et al. [90] employed pyrene-1-boronic acid, a glucose-sensitive material, in conjunction with rGO to functionalize the cooperative structural interface of p-type and n-type parallel/series connections, with the objective of achieving selective glucose detection. Pyrene-1-boronic acid possesses a conjugated π-electronic system that facilitates the formation of π-π interactions with graphene, thereby ensuring stable attachment to the graphene surface and enabling the detection of target analytes.

Side chain engineering is also a viable approach. Hao et al. [91] developed a heterojunction by integrating a carboxyl-functionalized two-dimensional (2D) polymer film with OSC. The activation of carboxyl groups on the 2D polymer film facilitated the formation of covalent bonds with prostate-specific antigen (PSA) antibody receptors. This film demonstrates a well-defined periodic structure that offers active sites for the immobilization of biological receptors, while exerting minimal effects on the semiconductor’s performance. Consequently, this configuration improves the sensor’s selectivity and sensitivity and mitigates the impact of non-specific adsorption.

The characteristics of OFETs can be modulated through precise regulation of the electric potential applied to the gate surface. Given the dynamic influence of the gate electric field on the charge carrier concentration within the channel, chemical sensing functionalities can be integrated at the gate interface [49]. In this context, Sasaki [49] immobilized glucose oxidase and an N-ethyl benzophenoneazine-based mediator onto the extended gate electrode via a SAM, enabling stable detection and analysis of analytes present in sweat. This approach also facilitates the straightforward determination of spermidine levels in cosmetic products, obviating the need for elaborate sample purification procedures [92].

The incorporation of components possessing specific recognition capabilities, achieved through techniques such as doping or interface engineering, is fundamental to imparting high selectivity to OFET sensors. Central to this approach is the transformation of non-specific physical adsorption into specific lock-and-key chemical interactions, thereby facilitating the precise identification of target analytes within complex matrices and enabling the progression from mere detection to selective discrimination.

### 3.3. Enhancing Stability and Reproducibility

Stability—including operational, environmental, and storage stability—is a critical bottleneck for the practical deployment of OFET sensors. Performance degradation often arises from trap-state generation at interfaces, electrochemical reactions with moisture or oxygen, or morphological changes in the active layer.

A common approach to improving device stability is dielectric interface modification. One study [93] systematically enhanced the performance and stability of n-type OFETs by introducing polyimide (PI) films of varying solid content onto an AlOx layer. The PI layer served as a charge-trapping layer whose internal dipoles aligned under the gate electric field, generating a stable built-in field that compensated for carrier trapping induced by gate-bias stress. This synergy of “interface trap regulation and dipole-field compensation” reduced interface trap density by 80% and significantly improved operational stability, with the drain current stabilizing after 1000 s of continuous bias stress. These results establish a solid foundation for the development of durable organic sensors.

To improve the electrical performance and long-term stability of OFET sensors, Wang et al. [38] introduced a double-layer structure composed of PAA:PEG and c-DPHA. This configuration effectively inhibits ion migration, decreases leakage current, prevents electrochemical doping, mitigates degradation of the semiconductor layer, enhances interface stability, and suppresses hysteresis effects.

Furthermore, incorporating 3 mol% of TeNF has been proposed as a means of achieving long-term stability and environmental robustness without encapsulation [94]. Due to its high electron affinity, TeNF preferentially captures injected electrons, preventing their conversion into hole traps and suppressing deep trap states. Simultaneously, TeNF fills polymer nanopores, reducing water-adsorption sites and minimizing the impact of moisture on sensing performance.

Innovations in device architecture may offer improved solutions. Viola proposed integrating an extended gate OFET with a liquid-processed ion-selective membrane. In this configuration, the extended gate structure physically isolates the sensing region from the OSC channel [95]. The ion-selective membrane, positioned atop the extended gate, facilitates specific interactions with the target analyte, and the resultant potential variations are transmitted to the distal OFET via gate capacitance. This design fundamentally mitigates the stability issues commonly encountered by OSCs when directly exposed to complex biochemical fluids, including electrochemical degradation, ion infiltration, and biological contamination. Consequently, this approach not only markedly enhances the device’s operational stability and longevity but also expands the potential applications of OFETs in challenging biochemical sensing environments, such as bodily fluid monitoring.

In summary, the optimization of stability constitutes a comprehensive engineering endeavor that necessitates collaborative design efforts. Whether it involves enhancing intrinsic stability through dielectric layer and molecular engineering, strengthening mechanical and environmental tolerance via blending and encapsulation, or implementing physical isolation through extended gate structures, the approaches aim to establish a durable and dependable protective encapsulation for the inherently delicate organic semiconductor core. Such advancements are essential for the transition of OFET sensors from experimental research settings to practical, real-world applications.

## 4. Discussion and Future Directions

This article summarizes various strategies for enhancing the performance of polymer OFETs through physical and chemical methods. The most efficient devices result not from a single approach but from the precise synergy between physical processing and chemical design. To facilitate a clearer comparison of the optimization strategies reviewed in this article, Table 2 summarizes the key points, organized by advantages, challenges, typical performance improvements, and contributions to sensor performance.

Polymers with good solubility and appropriate side chains form the foundation for effective physical processing. A non-crystallizable chemical structure, even under optimal annealing, shows limited improvement [96], whereas achieving optimal performance in D–A polymers requires optimizing their microphase separation through thermal annealing [59,62]. This synergistic effect is well demonstrated in the work of Ramer et al. [34], where both physical and chemical methods were combined to improve carrier injection and transport. Similarly, the study in [22] achieved substantial improvements in charge mobility by coordinating main-chain rigidity regulation, side-chain length optimization, and thermal annealing.

As reported by Jiang et al. [28], high device performance can be accompanied by excellent sensitivity, selectivity, and stability in wearable gas sensors, due to the high specific haodsurface area and capillary enrichment effects. Rather than relying solely on materials that maximize mobility, the study achieved function-oriented optimization through innovative device architecture.

However, enhancing carrier mobility frequently results in reduced on–off ratio, stability, or mechanical flexibility. Highly crystalline materials enhance μ but can also increase leakage current due to grain boundaries or film defects, thereby reducing the I_on_/I_off_ ratio [22], as demonstrated by Meresa et al. [88]. The use of dopants can enhance conductivity and mobility but may compromise the desired I_on_/I_off_ ratio. Furthermore, highly ordered films are more prone to cracking under mechanical stress, reducing flexibility and device reliability. No universally optimal method exists; rather, the device optimization strategy must be selected according to the specific application requirements.

The fundamental principle underlying blending and doping strategies involves the incorporation of a secondary phase to achieve properties unattainable by a single material; however, this approach frequently entails performance trade-offs. For example, the previously mentioned mixed porous material, UiO-66, served as an analyte concentrator [85]. Its large specific surface area and porous structure enabled pre-concentration of target gas molecules within the semiconductor layer, markedly increasing the likelihood and efficiency of gas–solid interactions. This improvement significantly lowered the LOD and increased sensitivity but at the expense of carrier mobility. This finding clearly illustrates that, in sensing applications, achieving extremely high sensitivity may necessitate deliberate trade-offs that sacrifice some degree of mobility.

Hemmi et al. [97] significantly improved the electrostatic imaging resolution and electrical performance of devices by constructing a one-dimensional array of floating-gate extended OFETs (FEG-OFETs) using printed electronics and semiconductor/polymer blending techniques. This application required not only sound electrical characteristics (such as adequate mobility and I_on_/I_off_ ratio) to detect weak signals, but also extremely high imaging resolution, charge-detection sensitivity, and long-term operational stability. While maintaining a carrier mobility of 0.67 cm^2^/V·s and an I_on_/I_off_ ratio of 10^4^–10^5^, the imaging resolution increased to 36.3 dpi, the minimum detectable charge density decreased to 30 pC/cm^2^, and the electric-field LOD reached 300 V/cm. Crucially, the synergistic effect between the deep-HOMO-level semiconductor and the parylene dielectric layer limited the Vth drift to 0.86 V within 6 h, successfully achieving an optimal balance between high electrical performance and excellent stability and sensitivity. This balance represents the key to success in electrostatic imaging applications.

Similarly, another study directly demonstrated the trade-off between generality and specificity. Researchers [98] used SAM surface modification to introduce different functional groups (trichloro(octyl)silane (C), N-[3-(trimethoxysilyl)propyl]ethylenediamine (2 N), N-[3-(trimethoxysilyl)propyl]ethylenediamine (3 N), and (3-mercaptopropyl)trimethoxysilane (S)) on silica particles to regulate their interaction with gas molecules, thereby achieving highly selective adsorption. Although some mobility was sacrificed, the response sensitivity to air increased fivefold compared with pure P3HT.

In future applications, electronic medical devices may incorporate not only a range of electrochemical sensors [49,92] but also biodegradable polymer semiconductors and dielectric layers designed to meet biocompatibility and degradability requirements [90]. Kulatunga et al. [99] used collagen as the dielectric layer due to its flexibility and biocompatibility comparable to human skin. It provided a relatively stable and suitable interface environment for the semiconductor layer, contributing to the flexibility and biocompatibility of the device.

By leveraging the short-range plasticity and memory effects of polymer OFETs, researchers aim to develop synaptic transistors for brain-like computing rather than focusing solely on high mobility [26,31,38,76,100,101]. Ding et al. [102] proposed a dual-gate OFET (DG-OFET), in which the polymer semiconductor channel was sandwiched between bottom-and top-gate dielectrics. This design enabled multiple electrical properties and synaptic behaviors on a single platform and supported analog logic processing. The top gate consisted of a ferroelectric copolymer, P(VDF-TrFE), while the bottom gate was SiO_2_. Through ferroelectric polarization and electrostatic doping effects, charge transport in the semiconductor layer was modulated, allowing precise regulation of device performance.

This approach also promotes semiconductor processing using aqueous or green solvents, reducing reliance on toxic organic solvents [67,103,104,105,106]. Hong et al. [107] prepared porous carbon materials via carbonization and mixed them with P3HT to fabricate OFETs. Particularly in samples carbonized with KOH, sensitivity was eight times higher compared with the original device, enabling high-performance sensing at low concentrations.

The development of polymer OFETs has shifted from performance-driven research to application-oriented design. Through deep synergy between chemical design and physical processing, a dynamic optimization network at the molecular–device interface level can be established, unlocking the full potential of polymer OFETs in emerging fields such as flexible sensing, bio-integrated electronics, and neuromorphic computing.

## Figures and Tables

**Table 1:** The influence of OFET performance parameters on sensing performance.

Performance of OFET	Performance of Sensors	Impact Trend	Underlying Mechanism	Ref.
μ	Sensitivity	**Positive correlation** The higher the value of μ, the higher the sensitivity.	High mobility implies higher sensitivity of channel conductance to changes in gate or channel surface potential.	[14,15,16]
	Response times	**Negative correlation** The higher the value of μ, the faster the response.	High mobility signifies faster charge carrier transport, allowing the channel current to reach a new equilibrium corresponding to the analyte concentration more quickly, thus speeding up the sensor’s response.	
	LOD	**Negative correlation** The higher the value of μ, the lower the LOD, the stronger the detection ability.	High mobility contributes to a higher signal-to-noise ratio and lower electrical noise, enabling the device to resolve minute current changes induced by very low analyte concentrations.	
Vth	Sensitivity	**Negative correlation** The lower the value of Vth, the higher the sensitivity.	For a device exhibiting stability and an appropriate initial Vth, the Vth shift caused by external stimuli is more easily detectable. Determining the operating voltage of the device, a lower Vth value is beneficial for the stability of the sensing signal. Conversely, instability in Vth drift generates noise and consequently impairs the LOD, resulting in a wrong judgment.	[16,17,18]
	Selectivity	The stability of Vth affects selectivity.		
	LOD	**Positive correlation** The lower the value of Vth, the lower the LOD.		
I_on_/I_off_	Sensitivity	**Positive correlation** The higher I_on_/I_off_ ratio, the higher the sensitivity.	A high I_on_/I_off_ ratio indicates a substantial range of current modulation between the “on” and “off” states. Devices exhibiting a high I_on_/I_off_ ratio within this extensive range are capable of generating a pronounced relative change in current with greater ease.	[17,18,19]
	LOD	**Negative correlation** The higher I_on_/I_off_ ratio, the lower the LOD.	A high I_on_/I_off_ ratio typically implies extremely low off-state current, which directly results in lower signal-to-noise ratio. This enables the device to distinguish minute changes in current signals caused by analytes with extremely low concentrations, thereby reducing the LOD.	
SS	Sensitivity	**Negative correlation** The smaller the value of SS, the higher the sensitivity.	The SS reflects the efficiency of gate voltage control over the channel current. The smaller the value, the more sensitive it is to the changes in interface charges or dipole moments caused by the analyte, which is beneficial for the detection of weak signals.	[17,18]
	LOD	**Positive correlation** The smaller the value of SS, the lower the LOD.		

**Table 2:** Comparison and summary of performance optimization strategies for OFET.

Strategies	Advantages	Disadvantages/Challenges	Typical Performance Improvement
Annealing	Simple and highly versatile process. It can increase crystallinity and molecular orderliness, reduce trap states, and enhance carrier transport.	The annealing temperature and time need to be precisely controlled. An excessively high temperature may disrupt the orderliness.	μ (***) Vth (**) I_on_/I_off_ (**) SS (**)
Solvent engineering	It can preset the film morphology at the initial stage of processing, compatible with solution processing, and construct a structure with a high specific surface area.	The selection of solvents and the optimization of their ratios are complex. The challenge of process reproducibility is significant.	μ (***) Vth (**) I_on_/I_off_ (**) SS (*)
SAMs	It can precisely control the interface energy level and chemical properties, effectively suppress interface traps, reduce interface traps, and enhance carrier injection and transport.	The quality of SAMs is highly dependent on the substrate, and the process is complex and potential long-term stability issues of SAMs.	μ (**) Vth (***) I_on_/I_off_ (**) SS (***)
Dielectric layer modification	The most direct approach for achieving low-voltage operation. It can optimize interface stability, reduce the operating voltage, and reduce leakage current.	High-k materials may introduce interface scattering and process compatibility issues.	μ (**) Vth (***) I_on_/I_off_ (**) SS (***)
Insertion of buffer layers	Effectively isolates the active layer from unfavorable interfaces. It can reduce RC, improve the electrode/semiconductor interface, and enhance carrier injection.	It may involve additional processing steps, thereby augmenting the complexity and cost of the procedure. The layer thickness and material selection are sensitive.	μ (**) Vth (**) I_on_/I_off_ (**) SS (*)
Main chain engineering	It can directly modulate the energy levels and molecular packing to attain enhanced mobility and stability. Fundamentally, it adjusts the intrinsic energy levels and the material’s capacity for charge delocalization.	The synthesis process is intricate and associated with elevated costs; moreover, establishing clear correlations between structure and properties can be challenging.	μ (***) Vth (**) I_on_/I_off_ (**) SS (/)
Side chain engineering	Effectively tunes solubility and molecular packing while preserving the backbone electronic structure.	Excessively long or bulky side chains can hinder charge transport. Additionally, they may introduce disordered and complex functional groups, potentially leading to a reduction in charge carrier mobility.	μ (***) Vth (**) I_on_/I_off_ (**) SS (*)
Blending	Straightforward and low-cost approach. It combines the advantages of multiple materials to deliver complementary functionalities, thereby enhancing both performance and stability concurrently.	It may reduce the mobility, and the control of phase separation is difficult.	μ (**) Vth (**) I_on_/I_off_ (**) SS (*)
Doping	It can substantially enhance conductivity, optimize contact interfaces, regulate carrier concentration, and decrease RC. Additionally, it serves as an effective method for tuning the Vth.	Difficult to precisely control concentration and distribution. It may introduce scattering centers, resulting in poor stability (easily prone to doping loss) and lead to reduced stability and increased off-current. It need high requirements for process control.	μ (**) Vth (***) I_on_/I_off_ (May be reduced) SS (/)

Note: / indicates no direct influence or an influence that can be disregarded. This strategy is not primarily aimed at this aspect, or there is no direct connection in terms of mechanism. * indicates a positive impact. It refers to the secondary or indirect effect of this strategy. ** indicates a significant impact. This is one of the core effects of this strategy. *** indicates a decisive/significant influence.

## Data Availability

Data supporting these findings are contained within this paper.
